# Investigation on Rational Utilization of Medicinal Plant *Semiliquidambar cathayensis* Chang Leaf and Bark at Different Developmental Stages

**DOI:** 10.3390/metabo15020098

**Published:** 2025-02-05

**Authors:** Juanling Li, Zhaopeng Geng, Yuanyuan Yuan, Minjuan Wang, Yanan Zhang, Junli Wang

**Affiliations:** 1 Key Laboratory of Ecology and Environment in Minority Areas (Minzu University of China), National Ethnic Affairs Commission, Beijing 100081, China; 21400266@muc.edu.cn (J.L.); 19400215@muc.edu.cn (Z.G.); 20400267@muc.edu.cn (Y.Y.); 21302328@muc.edu.cn (M.W.); 22302478@muc.edu.cn (Y.Z.); 2 College of Life and Environmental Sciences, Minzu University of China, Beijing 100081, China

**Keywords:** *Semiliquidambar cathayensis* Chang, medicinal plant, metabolomics, UPLC-MS/MS, rational utilization, different developmental stages

## Abstract

**Background:** *Semiliquidambar cathayensis* Chang is an extremely valuable and endangered medicinal plant. To investigate the exploitation and rational utilization of *S. cathayensis*, this study conducted metabolomics analysis of the leaves and bark of artificially cultivated *S. cathayensis* at different developmental stages. **Methods:** These metabolites were detected and identified by Ultra-Performance Liquid Chromatography–tandem Mass Spectrometry (UPLC-MS/MS) technology, and then univariate statistical analyses, multivariate pattern analyses, and pathway analyses were carried out. **Results:** As a result, a total of 801 metabolites were detected in *S. cathayensis*; differential metabolites in leaves at different developmental stages were mainly enriched in pathways related to flavonoids, whereas differential metabolites in bark at different developmental stages were mainly aromatic compounds, amino acids, and flavonoids, among others. This study revealed that young leaves are ideal for use in treating rheumatism, regulating blood pressure, and lowering blood glucose, while old leaves are better suited for skincare products and extracting materials to prevent neurodegenerative diseases and support women’s ovarian health. As for bark, four-year-old *S. cathayensis* bark is optimal for extracting myricetin. If the pharmaceutical, chemical, food, and industrial fields require extensive extraction of L-phenylalanine, trans-3-hydroxycinnamate, and 4-hydroxyphenylacetate, and if the medical field needs to extract anti-allergy, liver protection, and anti-coagulant ingredients, the two-year-old *S. cathayensis* bark is the best choice. **Conclusions:** Thus, this study established a solid theoretical framework for the rational, effective, and sustainable utilization of *S. cathayensis* leaves and bark.

## 1. Introduction

*Semiliquidambar cathayensis* Chang (Altingiaceae) is a species endemic to China, which has common characteristics with the *Liguidambar* and *Altingia* [1]. *S. cathayensis* is an extremely valuable and endangered medicinal plant, which was listed as a national key plant under Grade II protection by China in 1992 and was defined as a vulnerable plant by the International Union for Conservation of Nature [2]. *S. cathayensis* is mainly distributed in the mountainous regions of Southeast and South China [3]. The name and medicinal effects of *S. cathayensis* were first recorded in the *Lingnan Herbal Medicine Record* [4]. The roots, stems, leaves, bark, fruits, and even nectar of *S. cathayensis* all have valuable medicinal potential for exploitation. Specifically, its medicinal application varies according to different tissue parts and developmental stages [5].

As a traditional Chinese medicine, *S. cathayensis* has been used in China for thousands of years. *Chinese Materia Medica of Miao Medicinal Volume* has recorded that *S. cathayensis* root can be used to treat rheumatic and rheumatoid arthritis, foot swelling, lumbar and leg pains, migraine headaches, and paralysis [6]. In the past few decades, research has shown that the leaves of *S. cathayensis* have the functions of eliminating wind and stopping pain, unblocking meridians, and treating rheumatic pain as well as bleeding from traumatic injuries, while the bark can be used to treat anemofrigid–damp arthralgia, rubella, and skin itching [7,8]. Meanwhile, modern pharmacological investigations have confirmed that *S. cathayensis* has good anti-inflammatory, antioxidant, central analgesic, and anti-viral activities; it can also promote blood circulation and remove blood stasis [9]. These effects are attributed to the bioactive compounds contained in *S. cathayensis*, such as oleanolic acid, vitamins, flavonoids, isoflavones, triterpenoids, ellagic acid derivatives, resveratrol, polyphenols, glycosides, steroids, and other active compounds with pharmacological activity [10]. Studies demonstrated that oleanolic acid in *S. cathayensis* can effectively repair the necrotic areas of the liver and promote the recovery of normal function of liver tissues, which has significant liver protection effects [11]. Qiu et al. have also isolated quercetin, kaempferol-3-o-α-l-rhamnoside, kaempferol, 6′′-galloylmyricetin-3-o-β-d-glucopyranoside and gallocatechin from *S. cathayensis* leaf and indicated that all of these compounds showed strong tyrosinase inhibitory activity in a tyrosinase activity test [12]. There are also some studies showing that the polyphenols in *S. cathayensis* have strong antioxidant effects and can inhibit the growth of bacteria and cancer cells [8]. Furthermore, *S. cathayensis* is also an excellent ornamental tree commonly used in ecological afforestation and greening, and breakthrough progress has been made in the artificial breeding and cultivation of *S. cathayensis* [13].

However, previous studies indicated that wild *S. cathayensis* is an endangered medicinal plant, and it has poor ecological adaptability and weak natural regeneration ability [14]. Even so, the current methods used to pick and utilize wild *S. cathayensis* remain unsustainable. Additionally, in terms of effective and rational use, there has been little research on the exploration of a good deal of differential bioactive and effective compounds in *S. cathayensis* leaves and bark at different developmental stages and the underlying mechanism behind them, because during the development of plants, a series of changes in metabolite content in their bodies will occur due to the laws of their growth and development, as well as biotic and abiotic stresses brought by nature. Chlorophyll, for example, breaks down as leaves grow. Also, under high-temperature stress, in order to absorb UV radiation and protect themselves from damage, the content of total phenols and flavonols will increase in leaves. Additionally, drought stress can cause some young plants to produce more anthocyanins, etc [15]. Given this, researching artificially cultivated *S. cathayensis* can not only effectively further develop its differential bioactive compounds but also protect wild *S. cathayensis* resources to a certain extent. Therefore, based on criteria such as useful tissue, bioactive compound content, stability, and growth pattern, the young and old leaves of two-year-old artificially cultivated *S. cathayensis* and the bark of two-year-old and four-year-old *S. cathayensis* were selected in this study to conduct metabolomic analysis. The purpose of this study is to characterize the changes in metabolites in the leaves and bark of *S. cathayensis* at different developmental stages and analyze the potential underlying factors that contribute to these differences, facilitating the rational, effective, and sustainable utilization of *S. cathayensis*.

## 2. Materials and Methods

### 2.1. Chemicals and Plant Materials

Acetonitrile and methanol (LC-MS grade) were the products of CNW Technologies (Duesseldorf, Germany). Ammonium hydroxide (LC-MS grade) was supplied by Fisher Chemical (Waltham, MA, USA). LC-MS grade ammonium acetate was obtained from Sigma-Aldrich (St. Louis, MO, USA). Ultrapure water used throughout the experiments was obtained from the Milli-Q system ( Merck Millipore, Burlington, MA, USA). All the samples of artificially cultivated *S. cathayensis* in this experiment were collected from Hengshitang Town, Yingde, Qingyuan City, Guangdong Province, China (24°20 N, 113°21 E), on the morning of 14 May 2024 (sunny day). Among them, eight plants, each two-year-old and four-year-old, were selected. The same number of samples (30 g of old and young leaves from each two-year-old plant and 30 g of bark from each two-year-old and four-year-old plant) were taken from the same height above the ground in four directions: east, south, west, and north. It is important to note that when sampling, we collect old and young leaves according to the following criteria: We usually identify young leaves as bright green in color, with small leaves growing at the top of newborn branches or stems, and old leaves as darker in color, possibly darker green (due to the gradual degradation of chlorophyll in old leaves), and with larger leaves located at the lower part of the branches. Next, the samples were fixed by liquid nitrogen and transported to the laboratory. Each experimental treatment group consisted of samples of the same species collected in these four directions. Then, YD1 (young leaves of two-year-old *S. cathayensis*), YD2 (old leaves of two-year-old *S. cathayensis*), YD4 (bark of two-year-old *S. cathayensis*) and YD5 (bark of four-year-old *S. cathayensis*) were prepared for phytochemical analysis and metabolomics analysis.

### 2.2. Extraction and Preparation

The *S*. *cathayensis* sample (5 g) was accurately weighed and added to 50 mL of 85% methanol; the mixture was then subjected to ultrasound extraction at 40 °C for 30 min, followed by cooling to room temperature (25 °C). The extract was centrifuged at 4 °C for 15 min at a speed of 12,000× *g*. The resulting sample was filtered through a microporous filter membrane (Merck Millipore, Burlington, MA, USA)with a pore size of 0.22 μm, and then a volume of 200 μL from the filtered solution was absorbed. To prepare the sample for testing, an internal standard solution containing dichlorophenylalanine (2.9 g/L) in a quantity of 10 μL was added [16]. The preparation method of quality control (QC) samples is the same as above. The samples were then quantitatively analyzed by High-Performance Liquid Chromatography (HPLC) (Vanquish, Thermo Fisher Scientific, Darmstadt, Germany).

### 2.3. UPLC-MS/MS Analysis

An Ultra-Performance Liquid Chromatography (UPLC) system (Vanquish, Thermo Fisher Scientific, Darmstadt, Germany) equipped with a UPLC HSS T3 column (2.1 mm × 100 mm, 1.8 µm) was used for chromatographic analysis; the injection volume was 10 μL. Chromatographic conditions: column temperature was 40 °C, mobile phase A was ultrapure water (containing 0.1% formic acid), mobile phase B was ethyl acetate (containing 0.1% formic acid), flow rate was 0.3 mL/min, and sample size was 6 μL. Gradient elution procedures were as follows: 0~1.0 min, 5% solvent B, 1.0~6.0 min, 5~20% solvent B, 6.0~9.0 min, 20~50% solvent B, 9.0~13.0 min, 50~95% solvent B, 13.0~15.0 min, 95% solvent B, 15.0~18.0 min, 95~5% solvent B [17].

Mass spectrometry conditions: capillary voltage was 3.0 kV in positive ion mode; cone voltage was 40 V; mass scanning range was 50~1500 *m*/*z* (mass-to-charge ratio); mass spectra were collected every 0.2 s; for accurate mass determination, rutin solution (100 mg/L) was used as a locked mass solution; all parent ions were fragmented at energy of 20–40 eV; fragmented ion spectra were collected every 0.2 s.

### 2.4. Data Processing and Multivariate Pattern Analysis

The raw data from the analysis were converted to mzXML format with ProteoWizard(Version 3.1.0). Then, peak identification, extraction, alignment, and integration were carried out using a custom program developed in R based on the XCMS software package(Version 3.20.0). For metabolite annotation, the internal MS2 database (DB) known as BiotreeDB was used, which contains the reference spectra and information for known metabolites. Metabolites were annotated by comparing the obtained MS2 spectra with those in the database based on their similarity scores. An annotation threshold of 0.3 was set, meaning that if the similarity score of a metabolite exceeded this threshold, it was regarded as successfully annotated. Additionally, the identified metabolites were further analyzed and annotated via the Human Metabolome Database (HMDB) (https://www.hmdb.ca/ (accessed on 15 June 2024)). Principal component analysis (PCA) and orthogonal partial least squares discriminant analysis (OPLS-DA) were performed using *SIMCA* software (Version 16.0.2). To evaluate the OPLS-DA model, a 7-fold cross-validation was conducted, calculating R^2^ (model fitness) and Q^2^ (predictive ability), followed by 200 permutation tests [18]. Potential differential metabolites between groups were screened based on meeting both VIP >1 and *p* < 0.05 (*t*-test).

### 2.5. Metabolic Pathway Analysis

The metabolic enrichment pathways of potential differential metabolites involved in cell metabolism were analyzed by MetaboAnalyst (http://metpa.metabolomics.ca/ (accessed on 25 June 2024)) and the Kyoto Encyclopedia of Genes and Genomes (KEGG) pathway database (http://www.genome.jp/kegg/(accessed on 28 June 2024)) with the map of the model plant *Arabidopsis thaliana*. Through the comprehensive analysis of the pathways where the differential metabolites were located, the most important pathways that correlate with the metabolite difference were screened.

## 3. Results

### 3.1. Determination of Metabolites in S. cathayensis

**Figure 1:**
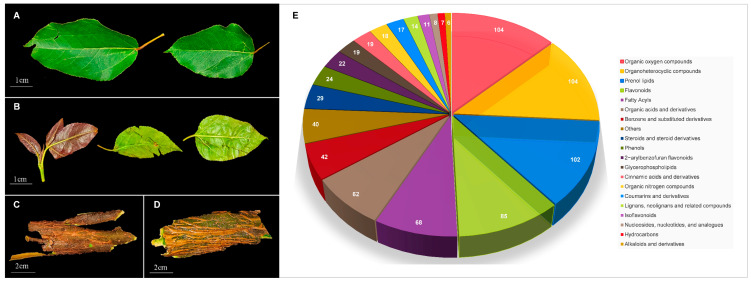
Determination of metabolites in *S. cathayensis.* (**A**) A photo of YD2. (**B**) A photo of YD1. (**C**) A photo of YD5. (**D**) A photo of YD4. (**E**) Classification of metabolites.

In the following Figure 1A–D, YD1 and YD2 are the young and old leaves of two-year-old *S. cathayensis*, and YD4 and YD5 are the bark of two-year-old and four-year-old *S. cathayensis*. A total of 801 metabolites were detected in the young and old leaves of two-year-old *S. cathayensis*, and the bark of two-year-old and four-year-old *S. cathayensis*, including flavonoids, isoflavones, organic acids, coumarins and their derivatives, alkaloids, terpenoids, phenols, pregnenolone ketones, benzene and its substituted derivatives, and so on (Figure 1E). The kind of metabolites contained in the four groups almost overlap, indicating that the differences between the samples of *S. cathayensis* were mainly reflected in the content rather than the kind of metabolites.

### 3.2. Identification of Differential Metabolites of S. cathayensis

**Figure 2:**
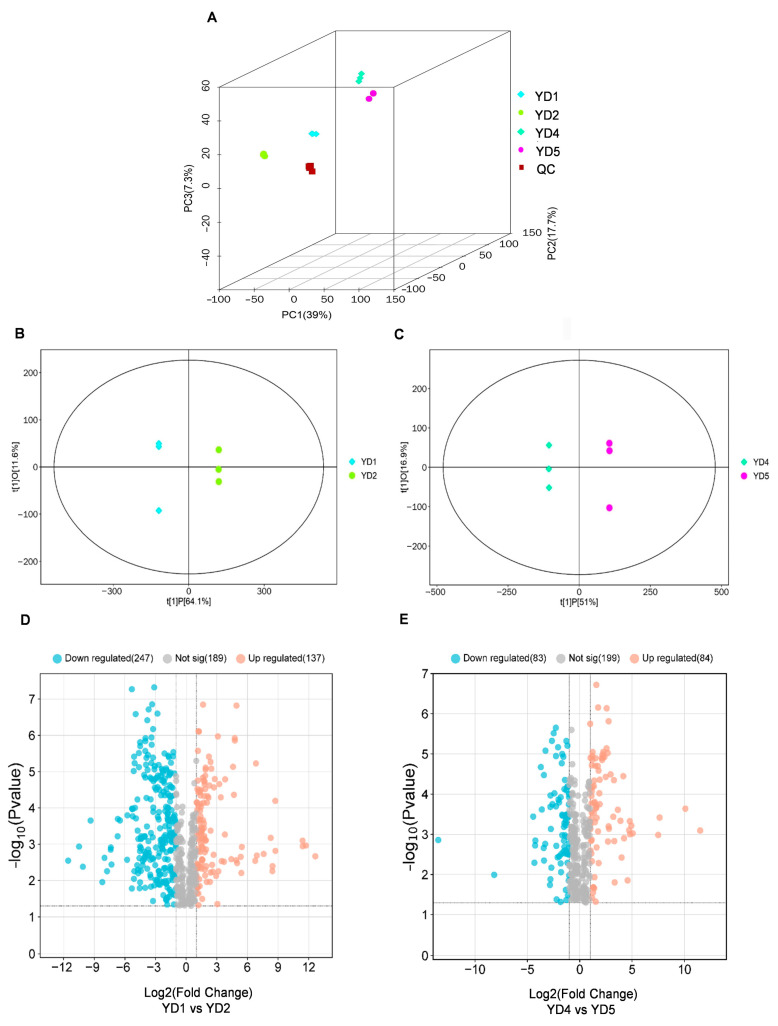
Metabolite profiles across the four groups of *S. cathayensis.* (**A**) PCA score map of total groups and QC. (**B**) OPLS-DA score map of YD1 and YD2. (**C**) OPLS-DA score map of YD4 and YD5. (**D**) Volcano map of YD1 vs. YD2. (**E**) Volcano map of YD4 vs. YD5.

**Figure 3:**
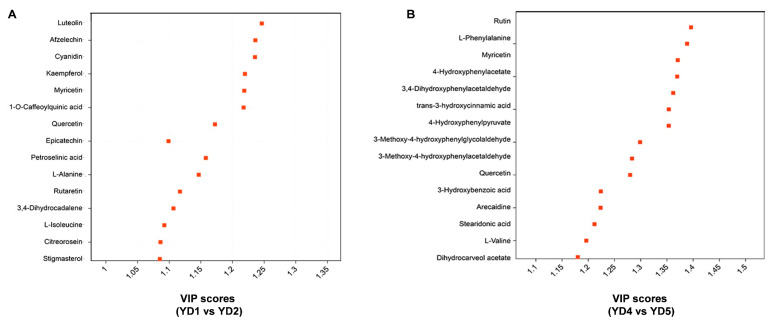
VIP scores map of the important metabolites with the top 15 VIP scores in each comparison group. (**A**) YD1 vs. YD2. (**B**) YD4 vs. YD5.

In Figure 2A, the biological replicates of the four samples gathered together separately in different areas and showed significant differences, among which the contributions of PC1, PC2, and PC3 in the figure to the separation of samples were 39, 17.7, and 7.3%, respectively. In order to find the effects of different developmental stages on metabolites in the same tissues of *S. cathayensis*, this research established OPLS-DA models, which found metabolic differences between YD1 and YD2, and between YD4 and YD5. As shown in Figure 2B,C, the score maps of the two models showed good separation between different groups. According to the high predictability of the OPLS-DA model (Q^2^) (Table S1), further verified by the permutation test (Figure S1), OPLS-DA models with good predictability and reliability were established. In this study, differential metabolites were screened according to VIP > 1 in OPLS-DA analysis and *p* < 0.05 in an independent-sample *t*-test, and the results are shown in Table S2. The screening results have been illustrated by a volcano plot (Figure 2D). Compared with YD2, there were 384 differentially expressed metabolites in YD1. Among them, 137 metabolites were up-regulated (luteolin and afzelechin, for example, were significantly up-regulated) and 247 metabolites were down-regulated (kaempferol and myricetin demonstrated a comparatively prominent down-regulation). Meanwhile, compared with YD5, 167 differentially expressed metabolites (84 up-regulated and 84 down-regulated) were screened out in YD4 (Figure 2E), for instance, some metabolites such as rutin, L-phenylalanine, and trans-3-hydroxycinnamate were significantly up-regulated, and myricetin was significantly down-regulated. The VIP scores of the important metabolites with the top 15 VIP scores in each comparison group are shown in Figure 3. In addition, the mean relative contents (the average of three repetitions) of each metabolite in each sample are shown in Table S2. As for the differential metabolites in flavonoid biosynthesis pathways that we focused on, the mean relative contents of luteolin and afzelechin in YD1 were 0.500 and 0.291, respectively, while those in YD2 were 0.045 and 0.028, respectively. Therefore, the relative contents of luteolin and Afzelechin in young leaves were 11.13 and 10.74 times those in old leaves; the mean relative contents of kaempferol and myricetin in YD1 were 16.539 and 0.779, and in YD2 were 30.475 and 1.176, respectively. Therefore, the relative contents of kaempferol and myricetin in old leaves were about 1.84 times and 1.51 times those in young leaves.

### 3.3. The Results of Metabolic Pathway Analysis

**Figure 4:**
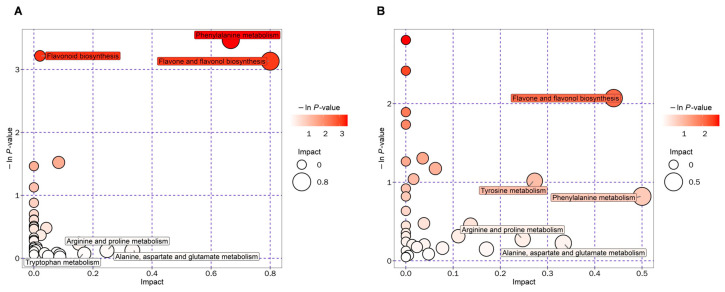
Pathway enrichment analysis of the differential metabolites. (**A**) YD1 vs. YD2. (**B**) YD4 vs. YD5.

In the KEGG database, the differential metabolites of the comparison of the leaf group and the bark group were analyzed for enrichment in metabolic pathways. The top 20 metabolic pathways were selected according to the influence values, such as *p*-value, the number of differential metabolites hitting the pathway (Figure S2), and the impact value of metabolic pathway topological analysis, which were listed in Tables S3 and S4. Then, the most important metabolic pathways were screened out, and the results were presented in the form of a bubble chart. As shown in Figure 4A,B, each bubble represents a pathway, and the abscissa and the ordinate represent the influencing factors of the pathway in the topological analysis. The larger the bubble, the darker the color, and the more important the pathway. After comprehensive consideration, the main enrichment pathways were marked: phenylalanine metabolism, flavonoid biosynthesis, and flavone and flavonol biosynthesis (in the comparison of the leaf group); phenylalanine metabolism, tyrosine metabolism, and flavone and flavonol biosynthesis (in the comparison of the bark group).

### 3.4. Recognition of Metabolite Differences in S. cathayensis Leaves at Different Developmental Stages

**Figure 5:**
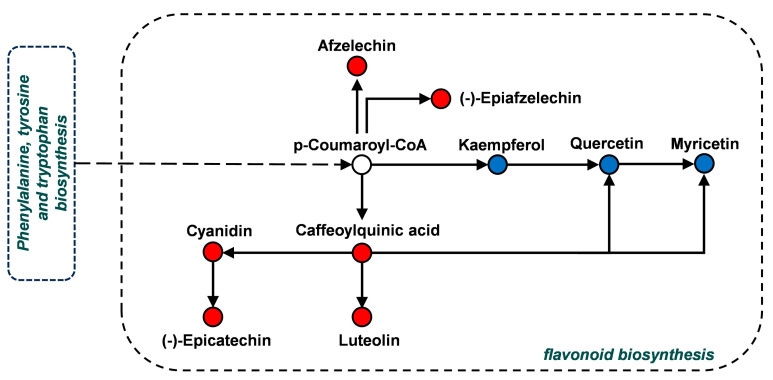
Flavonoid biosynthesis pathway in the comparison of YD1 and YD2 (blue dots represent down-regulated metabolites in YD1 when compared with YD2; red dots indicate up-regulated metabolites in YD1 when compared with YD2; solid arrows indicate biochemical processes within the same metabolic pathway, while dashed arrows indicate interpathway biochemical reactions).

As shown in Figure 5, the phenylalanine metabolic pathway, and flavonoid and flavonol biosynthesis were the main enrichment pathways for the differential metabolites in the comparison of YD1 and YD2. Among them, the phenylalanine pathway is necessary for the flavonoid synthesis pathway. Therefore, the difference between YD1 and YD2 was mainly reflected in the content of flavonoids.

**Figure 6:**
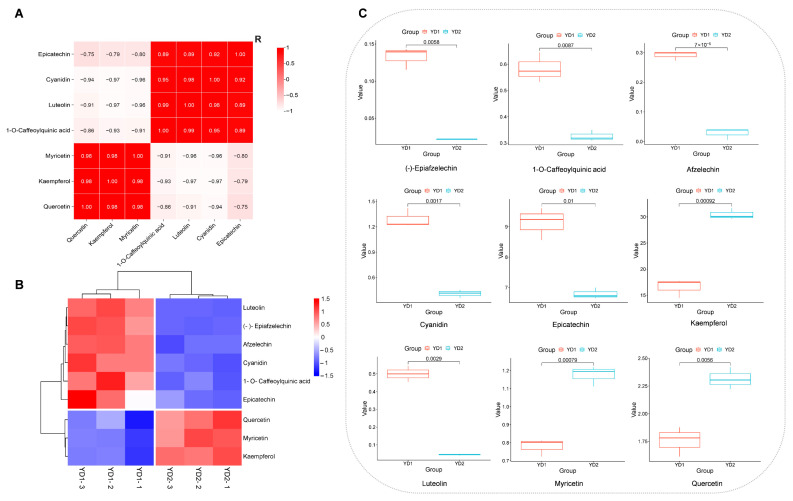
Data presentation related to flavonoid biosynthesis pathway. (**A**) Correlation heatmap for differential metabolites in the flavonoid biosynthesis pathway. (**B**) Hierarchical clustering heatmaps for differential metabolites in flavonoid biosynthesis pathway. The redder the color, the higher the comparative content of the metabolite, and the bluer the color, the lower the comparative content of the metabolite. (**C**) Box plot for differential metabolites in flavonoid biosynthesis pathway.

In the flavonoid biosynthesis pathway, p-coumaroyl-CoA obtained from the phenylpro panoid pathway can produce kaempferol, which can be converted into quercetin and then form myricetin. Additionally, p-coumaroyl-CoA can convert caffeoylquinic acid into cyanidin and then form epicatechin. Caffeoylquinic acid can also produce quercetin, myricetin, and luteolin (Figure 5). The relative contents of these differential metabolites in YD1 and YD2 are shown in Figure 6B,C. The relative content of kaempferol, quercetin, and myricetin was down-regulated in YD1 compared with YD2; in particular, kaempferol and myricetin were significantly down-regulated. Conversely, the relative content of afzelechin, epiafzelechin, caffeoylquinic acid, luteolin, cyanidin, and epicatechin was up-regulated in YD1 compared with YD2, with afzelechin and luteolin significantly up-regulated in YD1. In addition, the correlation of the relative content of metabolites is shown through a correlation heatmap (Figure 6A).

### 3.5. Identification of Metabolite Differences in S. cathayensis Bark at Different Developmental Stages

**Figure 7:**
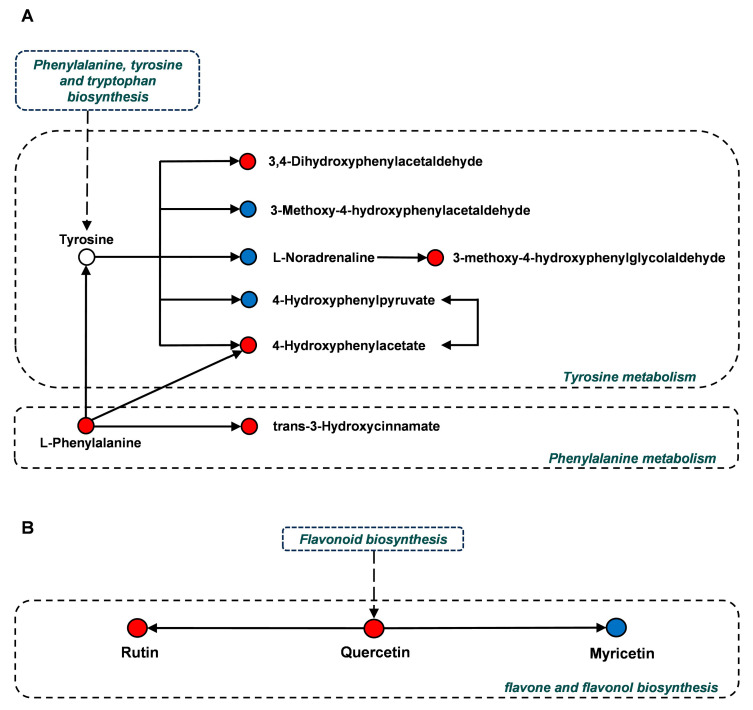
Phenylalanine metabolic pathway, tyrosine metabolic pathway, and flavone and flavonoid biosynthesis pathway in the comparison of YD4 and YD5. (**A**) Phenylalanine metabolic pathway and tyrosine metabolic pathway. (**B**) Flavone and flavonoid biosynthesis pathway. (blue dots represent down-regulated metabolites in YD1 when compared with YD2; red dots indicate up-regulated metabolites in YD1 when compared with YD2; solid arrows indicate biochemical processes within the same metabolic pathway, while dashed arrows indicate interpathway biochemical reactions).

**Figure 8:**
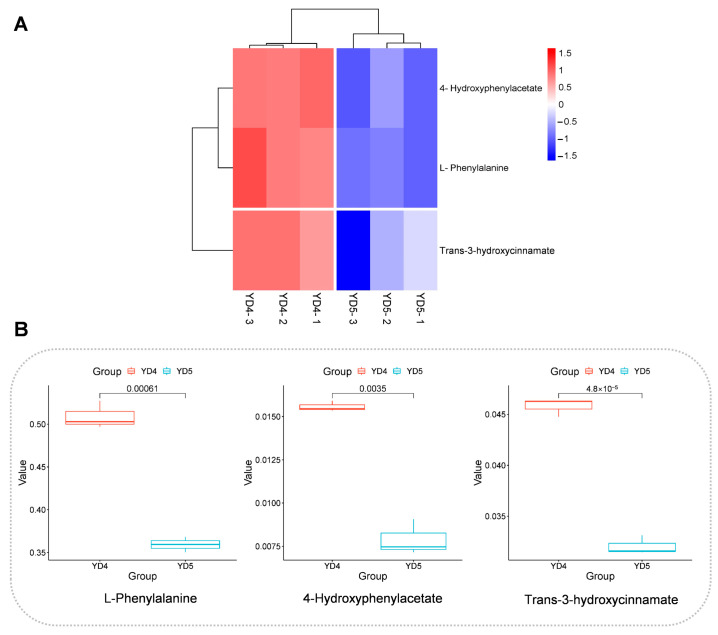
Data presentation related to phenylalanine metabolic pathway. (**A**) Hierarchical clustering heatmaps for differential metabolites in phenylalanine metabolic pathway. The redder the color, the higher the comparative content of the metabolite; the bluer the color, the lower the comparative content of the metabolite. (**B**) Box plot of differential metabolites in phenylalanine metabolic pathway.

**Figure 9:**
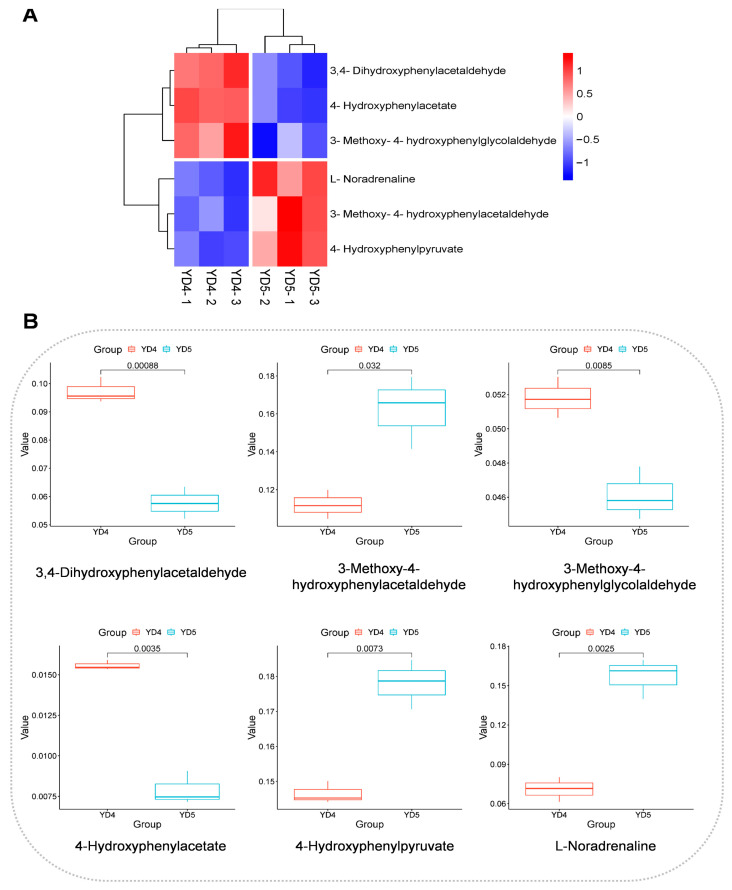
Data presentation related to tyrosine metabolic pathway. (**A**) Hierarchical clustering heatmaps of differential metabolites in tyrosine metabolic pathway. The redder the color, the higher the comparative content of the metabolite; the bluer the color, the lower the comparative content of the metabolite. (**B**) Box plot of differential metabolites in tyrosine metabolic pathway.

**Figure 10:**
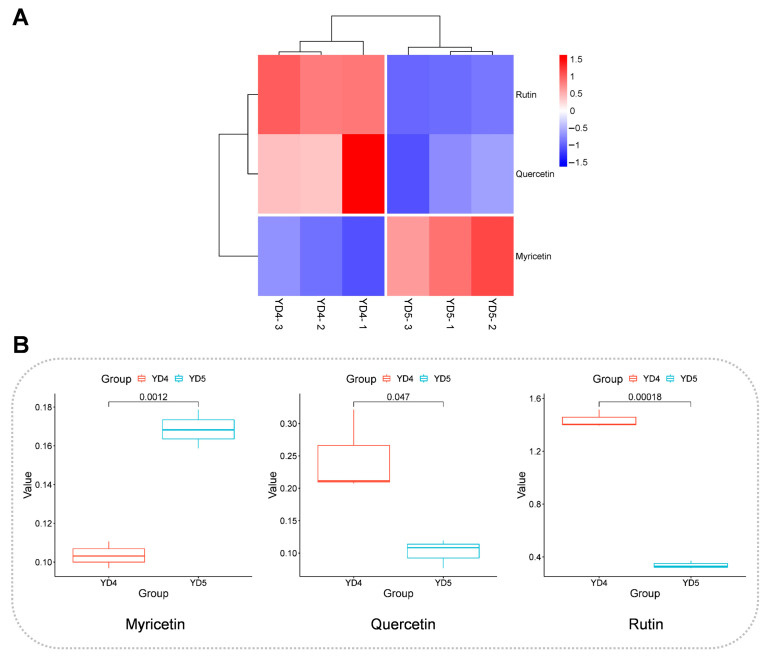
Data presentation related to flavone and flavonol biosynthesis metabolic pathways. (**A**) Hierarchical clustering heatmaps for differential metabolites in flavone and flavonol biosynthesis metabolic pathways. The redder the color, the higher the comparative content of the metabolite; the bluer the color, the lower the comparative content of the metabolite. (**B**) Box plot of differential metabolites in flavone and flavonol biosynthesis metabolic pathways.

In the enrichment analysis, pathways with significantly enriched differential metabolites between YD4 and YD5 were as follows (Figure 7A,B). Blue dots represent down-regulated metabolites in YD4 when compared with YD5; red dots indicate up-regulated metabolites in YD4 when compared with YD5. These pathways included phenylalanine metabolism, tyrosine metabolism, and flavonoid and flavonol biosynthesis pathways, among which the phenylalanine metabolism pathway and flavonoid and flavonol biosynthesis pathway were extremely closely related to flavonoid synthesis. In addition, the basic pathway of tyrosine metabolism is the synthesis pathway of tyrosine to synthesize tocopherol, plastoquinone, and panquinone, which is ubiquitous in plants and essential for plant growth and development. The hierarchical clustering heatmaps and the box plots of differential metabolites related to these important pathways are also shown in Figure 8, Figure 9 and Figure 10.

## 4. Discussion

### 4.1. Metabolite Differences in S. cathayensis Leaves at Different Developmental Stages

Phytochemicals vary with tissues and developmental stages in plant species. In this study, young and old leaves of two-year-old *S. cathayensis* planted artificially were selected. The differential metabolites between the two groups of leaves were predominantly phenylpropanoic acids and polyketides, especially flavonoids. The great difference in metabolic pathways highly associated with flavonoid synthesis in different growth stages of *S. cathayensis* leaves implied a significant difference in the accumulation of these compounds in different leaf periods.

Flavonoids are widely found in nature, and more than 8000 species have been identified in plants [19]. Recently, the functions of anthocyanins, flavonols, and flavanols in flavonoids have been intensively studied; for instance, modern medical research has concluded that flavanols possess the functions of regulating blood sugar, anti-inflammatory and antioxidant activities, and cardiovascular protection potential [20]. Meanwhile, in recent years, the phenomenon that the bioaccumulation of flavonoids exhibits species specificity, tissue specificity, and developmental specificity during the growth and development of plants has been revealed [21]. For example, Tian et al. found that the highest concentration of total flavonoid was in *S. cathayensis* leaves, while the contents of total flavonoid in *S. cathayensis* roots were low; they also discovered significant differences in the contents of total flavonoids in the leaves of *S. cathayensis* from different geographical origins [22].

In the comparison of YD1 with YD2, the old leaves had a longer growth period than young leaves and thus experienced more biotic and abiotic stresses, such as bird nibbling, high temperatures, and drought stress. Studies have shown that these stresses can also promote changes in the relative content of metabolites in plants. For example, under high-temperature stress, in order to absorb UV radiation and protect themselves from damage, the content of total phenols and flavonols will increase in old leaves, and drought stress can also cause some young leaves to produce more anthocyanins [15,23]. The materials of this experiment were taken from Yingde City, Guangdong Province (24°20 N, 113°21 E) in May, where the temperature is higher and the UV radiation is stronger. Therefore, to cope with and alleviate the damage received by the body, kaempferol, quercetin, myricetin, and other flavonols would accumulate in larger quantities in old leaves of *S. cathayensis* [24]. Among various phenolic flavonols, kaempferol, myricetin, and quercetin have strong antioxidant, neuroprotective, and skin-protective effects, and they play an important role in maintaining ovarian function and mitochondrial activity and restoring antioxidant defense enzyme activity [25]. Caffeoylquinic acid is an important flavonoid with diverse biological activities such as scavenging free radicals, chelating metal ions, modifying enzyme activity, and exerting anti-atherosclerosis, anti-mutagenesis, and anti-cancer effects [26]. Recent studies showed that the relative content of caffeoylquinic acid decreased with an increase in leaf area during leaf maturation, so it is likely that the rate of leaf growth surpassed the rate of caffeoylquinic acid synthesis, resulting in a lower caffeoylquinic acid content per unit of leaf fresh weight [27,28]. Moreover, the relative content of phenolic acid in plants generally decreases with an increase in leaf age during growth [28,29]. Consequently, the decrease in ellagic acid (a phenolic acid, a precursor to the synthesis of epicatechin) with leaf age is one of the most important reasons for the decrease in the relative content of epicatechin in old leaves [30]. Regarding the up-regulation of cyanidin in young leaves, studies have demonstrated that there were high concentrations of cyanidin-like substances in young plant leaves [31]. However, the concentration gradually decreased due to dilution and degradation as the leaf grew, resulting in changes to the green color characteristics of the leaf [32]. For this reason, as the growth of *S. cathayensis* leaves, the decrease in the relative content of cyanidin in them is partly attributed to the dilution of their concentration by the growth process and partly to their own degradation.

In addition, the correlation between metabolites can also be used to predict and explain the changes in their relative content in young and old leaves: when compared with old leaves, in young leaves, the down-regulation of quercetin and myricetin might be attributed to the down-regulation of kaempferol and the up-regulation of caffeoylquinic acid could result in the up-regulation of flavanols (luteolin, cyanidin, and epicatechin). The speculation can be further proved from the correlation heatmap in the previous part of this study, as the absolute values of the correlation coefficients of the seven compounds were all more than 0.74.

Based on the fact that the phenolic acids and flavanols in the young leaves of *S. cathayensis* were more abundant when compared with the old leaves, it can be inferred that the young leaves of *S. cathayensis* are more suitable for treating rheumatic and rheumatoid arthritis, regulating blood pressure and lowering blood glucose, and being made into health care products for these effects when compared with the old leaves. Meanwhile, due to the relatively higher content of flavonol in old leaves, old leaves are better suited for skincare products and extracting materials to prevent neurodegenerative diseases and support women’s ovarian health.

### 4.2. Metabolites Differences in S. cathayensis Bark at Different Developmental Stages

The analysis indicated significant differences in the relative content of compounds such as amino acids, aromatic compounds, and flavonols in YD4 and YD5.

This study demonstrated that L-phenylalanine, trans-3-hydroxycinnamate, and 4-hydroxyphenylacetate were up-regulated in YD4 compared to YD5. In particular, L-phenylalanine is an essential amino acid in living organisms and is critical for physiological functions and health. In addition, it is an important precursor for the synthesis of secondary substances such as coumarin, alkaloids, flavonoids, and lignin in plants [33]. It has been found that the relative content of L-phenylalanine in plants will gradually decrease with the growth and development process and is also influenced by the environmental temperature [33,34]. According to the results of this study, L-phenylalanine was up-regulated in YD4 compared to YD5. This phenomenon may not only be influenced by plant growth and development but may also be related to the fact that young *S. cathayensis* exhibited greater shade tolerance.

Trans-3-hydroxycinnamate is also an important secondary metabolite in plants with a variety of biological activities, such as antioxidant, anti-inflammatory, anti-bacterial, anti-tumor, hypoglycemic, and hypolipidemic [35]. In plant metabolism, trans-3-hydroxycinnamate is mainly an intermediate product of the phenylpropane metabolic pathway, which is generated from phenylalanine catalyzed by cinnamic acid hydroxylase and is involved in the synthesis of lignin and flavonoids [35]. Some studies have indicated that the plant of the genus *Maple* can release large amounts of anti-bacterial compounds such as 4-hydroxyphenylacetate, which has bacteriostatic effects on both itself and the surroundings [36]. And 4-hydroxyphenylacetate is an important raw material for the production of antipyretic and analgesic drugs [37]. In addition, it is also the main secondary metabolite of proanthocyanidins and kaempferol in polyphenols. In the current study, trans-3-hydroxycinnamate and 4-hydroxyphenylacetate were more abundant in two-year-old bark compared with four-year-old bark in *S. cathayensis*. This may be because young plants have lower immunity and require more nutrients and bacteriostatic compounds to maintain normal development. Overall, YD4 is a better choice compared to YD5 as L-phenylalanine, trans-3-hydroxycinnamate, and 4-hydroxyphenylacetate are needed for a wide range of extractions in the pharmaceutical, chemical, and food industries.

This study has found multiple differential metabolites in the tyrosine metabolic pathway by comparing YD4 and YD5, including 4-hydroxyphenylacetate, 4-hydroxyphenylpyruvate, 3-methoxy-4-hydroxyphenylacetaldehyde, 3-methoxy-4-hydroxyphenylglycolaldehyde, etc. Interestingly, almost all of these metabolites are involved in disease and stress resistance processes in plants. 4-hydroxyphenylpyruvate is directly related to the generation of plastoquinone and tocopherol in plant physiology, as well as having an effect on carotenoid and chlorophyll biosynthesis and lipid oxidation [38]. In clinical practice, 4-hydroxyphenylpyruvate is often used as one of the important biomarkers for the diagnosis of inborn metabolic disorders. In tyrosine metabolism, tyrosine is converted to 4-hydroxyphenylpyruvate (HPP) and glutamate by the catalytic conversion of tyrosine aminotransferase. Subsequently, HPP is converted to maleic anhydride acid (HGA) by p-hydroxyphenylpyruvate dioxygenase (HPPD) [39]. According to the results of some research, the enzyme activities in plants are correlated with their age and maturity [40]. Therefore, the relative content of 4-hydroxyphenylpyruvate was lower in YD4 than in YD5, potentially attributed to the lower tyrosine aminotransferase activity of YD4 compared to that of YD5. Moreover, 3-methoxy-4-hydroxyphenylacetaldehyde, which is also called coumarin, is commonly found in medicinal plants and is a compound with antioxidant, anti-inflammatory, anti-coagulant, and sedative effects [41,42]. In plants, coumarins (3-methoxy-4-hydroxyphenylacetaldehyde) can participate in the defense mechanisms of plants and promote the plants to fight against diseases and other organisms, such as pathogens and pests [43]. So, the reason why the relative content of coumarin in YD4 was lower than that in YD5 may be due to the fact that older trees need to focus more on long-term growth and resistance to diseases in terms of defense mechanisms than younger trees.

Through a comparative analysis, it was found that the main differential metabolites enriched in this pathway were quercetin, rutin, and myricetin. All three compounds belong to the flavonoids, and all of them have a variety of medicinal bioactivities, such as antioxidant, anti-bacterial, anti-viral, anti-sensitizing, anti-inflammatory, and anti-tumor properties, and cardiovascular and nervous system protection. Among them, quercetin and rutin can better inhibit histamine release and relieve allergies; rutin can also improve blood circulation and protect the liver [44,45]. Among these compounds, quercetin is a common compound found in a variety of plants and is especially abundant in the bark. It has been demonstrated that the accumulation of metabolites in some medicinal plants, such as chlorogenic acid, rutin, hyperin, and quercetin, varies greatly with the age of trees and that these compounds are mainly found in the bark of relatively young trees [39]. This corroborates the results of this study. Myricetin is a natural compound with significant anti-cancer activity. Myricetin exhibited in vitro cytotoxicity against a variety of human cancer cell types, including human cervical cancer HeLa cells, human melanoma A375-S2 cells, human breast cancer MCF-7 cells, and human hepatocellular carcinoma HepG2 cells [46,47].

Overall, in the field of biopharmaceutical applications, YD5 is the best choice for the extraction of myricetin compared to YD4. If the pharmaceutical, chemical, food, and industrial fields require extensive extraction of L-phenylalanine, trans-3-hydroxycinnamate, and 4-hydroxyphenylacetate, and if the medical field needs to extract anti-allergy, liver protection, and anti-coagulant ingredients, YD4 is the best choice. In the future, if possible, our research needs to explore the metabolic differences of more organs in *S. cathayensis* in different developmental stages to obtain more complete research results.

## 5. Conclusions

In conclusion, the aim of this study was to find the difference in *S. cathayensis* leaves and bark at different developmental stages to explore the method of their rational utilization. The results revealed that phenolic acids and flavonols were more abundant in the young leaves compared to old leaves of *S. cathayensis*. Notably, the relative contents of luteolin and Afzelechin in young leaves were 11.13 and 10.74 times those in old leaves, which makes them more suitable for further use in treatment of rheumatism and rheumatoid arthritis, blood pressure regulation, and blood glucose lowering. Meanwhile, kaempferol and myricetin in old leaves were about 1.84 times and 1.51 times those in young leaves, so the old leaves are better suited for skincare products and extracting materials to prevent neurodegenerative diseases and support women’s ovarian health. As for bark, four-year-old *S. cathayensis* bark is optimal for extracting myricetin. If the pharmaceutical, chemical, food, and industrial fields require extensive extraction of L-phenylalanine, trans-3-hydroxycinnamate, and 4-hydroxyphenylacetate, and if the medical field needs to extract anti-allergy, liver protection, and anti-coagulant ingredients, the two-year-old *S. cathayensis* bark is the best choice. This research established a solid theoretical framework for the rational, effective, and sustainable utilization of *S. cathayensis* leaves and bark and could also maximize the commercial cultivation potential of artificially cultivated *S. cathayensis.*

## Data Availability

The original contributions presented in this study are included in the Supplementary Material. Further inquiries can be directed to the corresponding author.

## Supplementary Materials

The following supporting information can be downloaded at https://www.mdpi.com/article/10.3390/metabo15020098/s1. Figure S1: Permutation tests of the OPLS-DA models; Figure S2: The top 20 metabolic pathways enriched by differential metabolites; Table S1: Parameters of OPLS-DA model; Table S2: Detected metabolites and differential metabolites in *S. cathayensis*; Table S3: The top 20 metabolic pathway analysis (YD1 vs. YD2); Table S4: The top 20 metabolic pathway analysis (YD4 vs. YD5).
