# Supplementary material for: Investigation on Rational Utilization of Medicinal Plant Semiliquidambar cathayensis Chang Leaf and Bark at Different Developmental Stages
Source: Metabolites. 2025 Feb 5;15(2):98. doi: 10.3390/metabo15020098 (PMC11857821; doi:10.3390/metabo15020098)
Supplement: Supplementary file 1 [file metabolites-15-00098-s001.zip › SI-Figures.pdf]

Supplementary figures for:

Investigation on rational utilization of medicinal plant *Semiliquidambar cathayensis* Chang leaf and bark at different developmental stages

Juanling Li [1,2], Zhaopeng Geng [1,2], Yuanyuan Yuan [1,2], Minjuan Wang [1,2], Yanan Zhang [1,2] and Junli Wang [1,2,*]

[1]Key Laboratory of Ecology and Environment in Minority Areas (Minzu University of China), National Ethnic Affairs Commission, Beijing 100081, China

[2]College of Life and Environmental Sciences, Minzu University of China, Beijing 100081, China

*Correspondence: junliwang@muc.edu.cn; Tel.: 86-010-68932633

Table of Contents

Supplementary Figure S1 Permutation tests of the OPLS-DA models. (A) YD1 vs YD2. (B) YD4 vs YD5.

Supplementary Figure S2 The top 20 metabolic pathways enriched by differential metabolites. (A) YD1 vs YD2. (B) YD4 vs YD5.

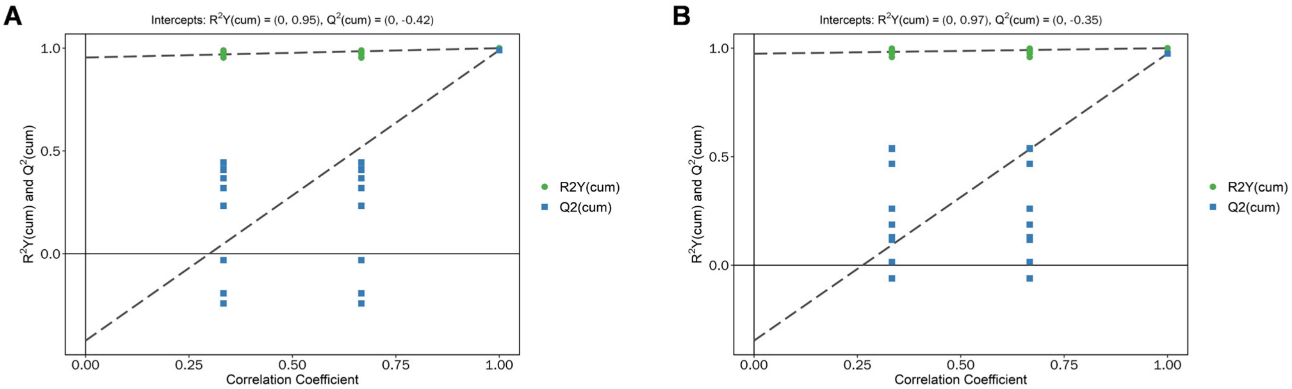

Figure S1. Permutation tests of the OPLS-DA models. (**A**) YD1 vs YD2. (**B**) YD4 vs YD5.

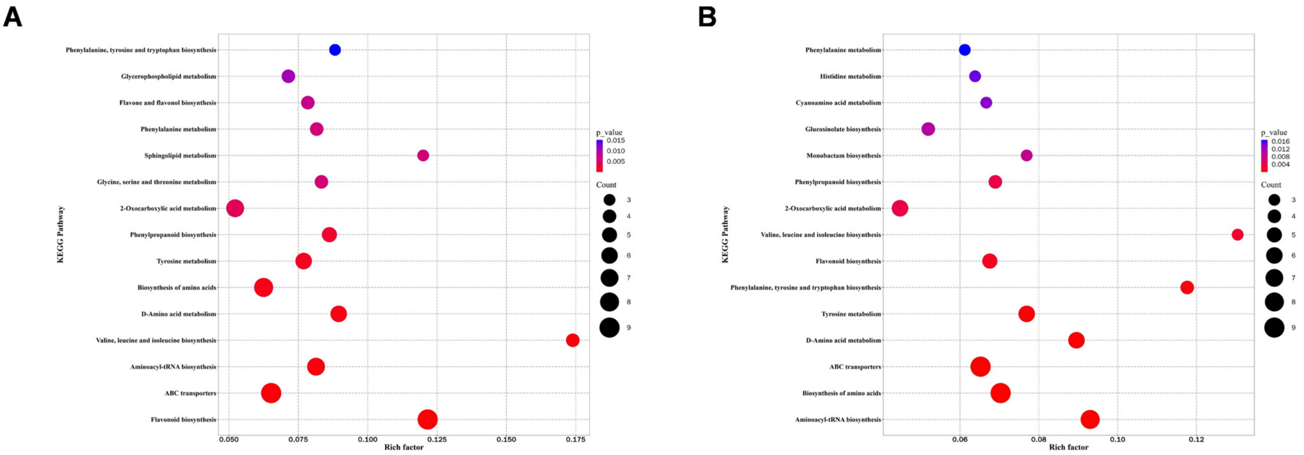

Figure S2. The top 20 metabolic pathways enriched by differential metabolites. (**A**) YD1 vs YD2. (**B**) YD4 vs YD5.
